# Medical Opinion and Movement

**Published:** 1909-02-27

**Authors:** 


### Medical Opinion and Movement.

Dr. H. Grabower, of Berlin, has applied the method of passive hyperæmia for the relief of the dysphagia accompanying laryngeal tuberculosis. For this purpose he uses an indiarubber band 2½ cm. wide, on the middle and on the inner side of which is attached a convex pad, which fits into the suprasternal fossa. In order to fix this collar bands may also be adapted to pass under the armpits. The collar should be applied below the larynx between the lower border of the cricoid cartilage and the upper margin of the clavicles, and it must be drawn as tight as the patient can bear it. He states that the patient soon gets accustomed to the collar and is able to sleep wearing it. The collar should be left on for six consecutive hours on the first day, twelve hours on the second, eighteen on the third day, and twenty-two hours afterwards. At first the œdema is increased, but the tenderness to touch and pressure much diminished. After three or four days the patient can swallow food, even solids, and at the end of 10 or 12 days the infiltration has more or less disappeared and there is no pain on swallowing. The collar should then be worn every third or fourth day, otherwise the dysphagia is likely to return, but it seems possible that after prolonged treatment for several weeks the band could be dispensed with for longer intervals.

Dr. Schnitzler draws attention to a metastatic growth which not infrequently accompanies cancer of the stomach, namely, cancer of the rectum. He has observed eleven cases, and gives the following characteristics of this metastatic growth. It occupies the anterior wall of the rectum just above the prostate. It is interstitial, and the mucous membrane is stretched over it and fixed, but not ulcerated, and it does not bleed. It is, therefore, quite different from the primary cancer of the rectum which is always epithelial, ulcerated, and bleeds readily. Although there is no ulceration in the early stages it may in the process of growth eventually open into the rectum, when it may first be recognised. It is important, therefore, in cases of cancer of the stomach to bear in mind the possibility of a metastasis in the rectum, and to include this fact in the general survey of the body for signs of secondary deposits. In cases, too, in which a gastric cancer may only be suspected, the discovery of a rectal growth would offer valuable evidence. It is necessary also to bear in mind the distinguishing features between a primary and secondary rectal growth. Dr. Schnitzler records the case of a rectal growth for which he removed the rectum, only to discover later that it was secondary to a gastric cancer which had hitherto remained latent.

According to the reports of Dr. Lehrmitte and Dr. Beaujard we may look to the *x*-rays as a therapeutic means of considerable value in the treatment of syringomyelia. They have treated several cases by subjecting the spine to the effect of the rays, and in all marked improvement has followed. Even in cases of long-standing radiotherapy is able to effect an improvement in certain symptoms and to diminish the most painful manifestations of the affection. Improvement always takes place in the same chronological order. Motor troubles are first affected and power reappears in the muscles where the weakness was most marked, vicious attitudes are in part corrected, whereas the amyotrophy is but little changed. Then the sensory symptoms undergo change for the better, tactile anæsthesia disappears, and a little later the thermo-analgesia diminishes in extent and in intensity. Even trophic affections of the bones and skin show the beneficent effect of the treatment. Moreover, it has been recently pointed out that radiotherapy may also afford assistance in the diagnosis of the disease. It is not infrequent for the disease to be confounded with leprosy, and a differential diagnosis in certain cases may offer considerable difficulty. Leprosy also often yields in a marked manner to radiotherapy, but in quite a different way. In this disease the *x*-rays must be applied to the peripheral nerves of the affected part, whereas in syringomyelia, as already stated, they are applied to the spinal cord. In leprosy no effect is produced by the action of the rays on the cord, and in syringomyelia no good appears to result from the application of the rays to the peripheral nerves. Further, in syringomyelia if treatment be limited to the upper segment of the cord, the corresponding part of the body is alone affected; on the other hand, in leprosy local treatment with the rays not only may effect a cure of the part treated but also amelioration of the disease in other parts.

It has already been shown by experimentation on animals that when an animal has been subjected to a series of bleedings the serum of that animal contains certain co-called hæmopoïetic substances, and when this serum is injected into some other animal it excites the hæmopoïetic organs and causes a considerable increase in the number of red blood corpuscles. Dr. P. Carnot has carried out a good deal of work on these lines, and finds that with rabbits the injection of five cubic centimetres of such a serum produces an increase of one to three million corpuscles per cubic millimetre in two or three days. This excess of corpuscles diminishes slowly, but persists for one or two months. He has applied the method to cases of anæmia and has obtained the same results in the human subject as in animals. He finds that unheated antidiphtheritic serum answers well for the purpose if it is taken from a horse that has been bled systematically at intervals. Dr. Carnot has used the dried antidiphtheritic serum obtained from the Pasteur Institute at Lille. Here the immunised horses are bled every two or three weeks to the extent of five or six litres. Moreover, the method can be further simplified by allowing the patient to take the serum by the mouth, either dried in cachets or liquid in the usual vials. Two to four grammes or 10 to 20 c.c. may be given in this way each day till the desired effect is obtained. No untoward symptoms have resulted from the ingestion of the serum in this way.

